# The Prevalence of *virB2* and *16SrRNA* Genes of *Brucella Isolated* from Clinical Samples of Hospitals, Western Iran

**Published:** 2018-09

**Authors:** Rahim SOROURI, Shoaib KHADAMORADI, Azad KHALEDI, Amir Hossein ABADI, Davoud ESMAEILI

**Affiliations:** 1. Applied Microbiology Research Center, Microbiology Department, Baqiyatallah University of Medical Sciences, Tehran, Iran; 2. Antimicrobial Resistance Research Center, Avicenna Research Institute, Dept. of Microbiology and Virology, Faculty of Medicine, Mashhad University of Medical Sciences, Mashhad, Iran; 3. Dept. of Microbiology and Immunology, Faculty of Medicine, Kashan University of Medical Sciences, Kashan, Iran

## Dear Editor-in-Chief

Brucellosis is a zoonotic disease and its incidence arises in most cases from a direct or indirect contact with infected animals. Brucellosis is an occupational disease that often threatens ranchers, veterinarians, slaughterhouse workers, and laboratory personnel (1).

Prior to 2001, in order to identify *Brucella* in patients, biochemical tests were used. Then *B. abortus16SrRNA* was used as a detection method for *Brucella* in 2001 by determining the variable sequences of these molecules that were specific markers for genus or species (2). The disadvantages of detection methods using culture and biochemical tests are the seven-day time for the result, and also the risk relating to working with *Brucella* in vitro. However, these complications were resolved using the sequence analysis of *16SrRNA* (3).

Operon VirB constitutes the Type IV secretory system (T4SS) which has an important role in the proliferation of intracellular bacteria and is one of the significant virulence factors of *Brucella*. VirB operon is required for intracellular growth and creating infection (4). Among the components of the operon virB, VirB 2 protein has attracted more attention. It is located on the bacterial surface and provides a channel for proteins or factors secreted by the bacterium in an external environment (5). No evidence is available about the frequency of *virB2* in *Brucella* spp., and the importance of this protein in the operon virB.

So, we aimed to evaluate the prevalence of genes*; virB2* and 1*6SrRNA* of *Brucella* in clinical samples achieved from hospitals in the west of Iran.

One hundred serum samples were collected from patients referred and admitted to hospitals of the Kermanshah Province, western Iran in 2014. These samples were positive as for Wright test (>1/80). Sequences of the virB2 gene and 16SrRNA were obtained from the NCBI and the primers designed using proper software. The primers sequences were as follows: F-16s: 5-CAGAGTGCAATCCGAACTGA-3, R-16s: 5-CAGCTCGTGTCGTGAGATGT-3, F-virb2: 5-TCTTGGATCCCATCTTCAGG-3, R-virb2: 5-GGGCTTCAATCCTTATGCAA-3. The PCR technique in this study is as follows: primary denaturation: 94 °C for 5 min, denaturation at 90 °C for 1 min, annealing at 58 °C for 1 min, extension step: 72 °C for 1 min, 35 cycles and final extension: 72 °C for 5 min. A Commercial DNA kit (Cinnagen Company) was used for extracting DNA from the serum samples. PCR was performed according to the guidelines. Finally, for evaluating the PCR results, electrophoresis was carried out. The data were analyzed using the SPSS software (Version 18; SPSS Inc., Chicago, USA).

**Fig. 1:**
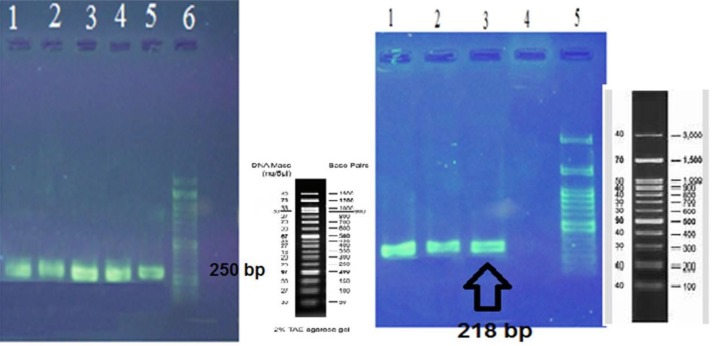
Left image; Results of products electrophoresis with 16SrRNA primers. Wells 1–5. Examples of positive samples for *16SrRNA* gene and well 6 are related to DNA marker 100bp. Right image; Results of products electrophoresis with virB2 primers. Wells 1, 2 and 3 are examples of positive samples for *virB2* gene, well 4 shows the negative control and the well 5 is related to DNA marker 100bp

Among 100 clinical isolates of *Brucella*, 37 cases (37%) had *16srRNA* gene and 33 isolates (33%) had *virB2* gene (Fig. 1). In this investigation, 33% of *Brucella* isolates contained the *virB2* gene. This finding leads to the conclusion that the secretory system has a role in intracellular survival and chronic infection. Today, one of the problems in the treatment of chronic *Brucella* infection comes from a lack of accurate identification in the laboratory by serological tests.

The merit of this research lies in the fact that *virB2* is considered as a candidate for identification of T4SS system of *Brucella* isolates. The other good characteristic of this research is that the frequency of this gene is determined from 100 clinical samples.

This study investigated for the first time the role played by the T4SS in serum samples. In order to specific study of *Brucella*, *virB2* gene should be used concurrently of specific gene and it’s sequencing together *16SrRNA*. This study used for the first time the *virB2* gene for diagnosis of *Brucella* in the serum. Thus, the virB2 gene can be used as a diagnostic factor along with other components.
